# A Drug Repositioning Approach Identifies a Combination of Compounds as a Potential Regimen for Chronic Lymphocytic Leukemia Treatment

**DOI:** 10.3389/fonc.2021.579488

**Published:** 2021-05-28

**Authors:** Atef Nehdi, Nosaibah Samman, Abdullah Mashhour, Alshaimaa Alhallaj, Thadeo Trivilegio, Sheraz Gul, Jeanette Reinshagen, Ahmed Alaskar, Gamal Gmati, Khadega A. Abuelgasim, Fatmah Mansour, Mohamed Boudjelal

**Affiliations:** ^1^ Medical Research Core Facility and Platforms, King Abdullah International Medical Research Center, Riyadh, Saudi Arabia; ^2^ Department of Life Sciences, Faculty of Sciences of Gabes, University of Gabes, Gabes, Tunisia; ^3^ King Saud bin Abdulaziz University for Health Sciences, Riyadh, Saudi Arabia; ^4^ Fraunhofer Institute for Molecular Biology and Applied Ecology IME-ScreeningPort, Hamburg, Germany; ^5^ Division of Hematology & HCT, Department of Oncology, King Abdulaziz Medical City, Riyadh, Saudi Arabia

**Keywords:** CLL, fludarabine, Isoprenaline, ATP depletion, synergistic effect, Ibrutinib, BTKi

## Abstract

Drug repositioning is a promising and powerful innovative strategy in the field of drug discovery. In this study, we screened a compound-library containing 800 Food and Drug Administration approved drugs for their anti-leukemic effect. All screening activities made use of human peripheral blood mononuclear cells (PBMCs), isolated from healthy or leukemic donors. Compounds with confirmed cytotoxicity were selected and classified in three groups: i) anti-neoplastic compounds which are drugs used in leukemia treatment, ii) compounds known to have an anti-cancer effect and iii) compounds demonstrating an anti-leukemic potential for the first time. The latter group was the most interesting from a drug repositioning perspective and yielded a single compound, namely Isoprenaline which is a non-selective β-adrenergic agonist. Analysis of the cytotoxic effect of this drug indicated that it induces sustainable intracellular ATP depletion leading, over time, to necrotic cell death. We exploited the Isoprenaline-induced intracellular ATP depletion to sensitize primary leukemic cells to fludarabine (purine analogue) and Ibrutinib (Bruton’s tyrosine kinase inhibitor) treatment. *In-vitro* treatment of primary leukemic cells with a combination of Isoprenaline/fludarabine or Isoprenaline/Ibrutinib showed a very high synergistic effect. These combinations could constitute a new efficient regimen for CLL treatment following successful evaluation in animal models and clinical trials.

## Introduction

Drug repositioning, also known as drug re-tasking, drug rescuing, therapeutic switching, drug recycling, drug repurposing or drug re-profiling, constitutes an alternative approach to the traditional methods of drug discovery, which, in addition to being extremely costly and time consuming, is a high-risk process. Drug repositioning has increasingly been used to streamline drug development for clinical use (1). As stated in a Food and Drug Administration (FDA) report (2016), the number of drugs approved by the FDA has been declining since 1995 (2). For oncology, the situation is even more challenging with the approval rate for new drugs at least 50% lower than for other indications (3). New approaches are therefore required to expedite the inherent risks associated with drug discovery. The starting point for drug repositioning is having access to a library of approved drugs. As the safety, efficacy and toxicity of these drugs have been extensively investigated resulting in FDA approval, identifying an additional use for these drugs offers the potential to accelerate the drug discovery process, thereby saving time and money (4, 5). The cost of the drug repositioning approach could be ten-fold lower than developing a new drug which is estimated at $1.6 billion (6) which offers many countries an opportunity to develop drugs with reduced investment. Additional advantages of the drug repositioning approach is the 30% approval rate compared to 10% for *de-novo* drug discovery (5).

One of the main reasons for the unusually high clinical failure rate of potential drugs in cancer research is the use of cancer cell lines as cancer models. Human cancer-derived cell lines are widely used in laboratories to study the biology of cancer, and to test the therapeutic efficacy of anti-cancer agents (7). However, the clinical relevance of cancer cell lines as a cancer model has always been questionable. Cancer cell lines poorly represent the diversity, heterogeneity and drug-resistance of tumors occurring in patients (8). Pre-clinical drug development has traditionally relied on established human cancer cell lines, cultured in serum-containing media in adherent conditions for extended periods. The *in-vitro* culture conditions, under non-physiological concentration of many metabolites and growth factors, can result in the accumulation of significant numbers of mutations and chromosomal aberrations (9, 10). Therefore, these genetically altered cancer cell lines do not accurately represent the clinical scenario (11).

The refinement of pre-clinical immortal cancer lines and use of patient derived primary cancer cell lines for clinical outcomes offers the potential to reduce the clinical attrition rates of cancer therapeutics. A major hurdle to drug screening using primary cancer samples is their availability, accessibility and the low sample input, consequently these cell types are not considered as a viable model for cancer drug screening (12). Primary cancer cell lines are mainly used in precision medicine to predict the clinical activity of cancer drugs in individual patients (13, 14). However, in contrast to solid tumors, leukemia patients provide an abundant source of primary cancer cells, and therefore are a suitable model for leukemic drug screening.

In the present study, we performed phenotypic screening on primary leukemic cells against a library of FDA approved drugs. Primary leukemic cells were isolated from blood samples derived from patients with Acute Myeloid Leukemia (AML), Acute Lymphocytic Leukemia (ALL), Chronic Lymphoid Leukemia (CLL), Chronic Myeloid Leukemia (CML) and from four healthy donors. Cell viability was determined using the CellTiter-Glo luminescence-based phenotypic assay which led to the identification of 31 compounds which were associated with activity in at least one of the tested leukemia subtypes. These compounds were classified in three groups: i) anti-neoplastic compounds used in leukemia treatment, ii) compounds known to have an anti-leukemic effect and iii) compounds demonstrating an anti-leukemic potential for the first time. This latter group contained three compounds: silver sulfadiazine, chlorhexidine and Isoprenaline. Since the two first are approved for only external use, Isoprenaline was selected for further pre-clinical characterization.

## Materials and Methods

### Study Participants and Ethical Consideration

The study protocol was approved by the Institutional Review Board of King Abdullah International Medical Research Center and all methods were performed in accordance with the World Medical association declaration of Helsinki on ethical principles for medical research involving human subjects. All donors recruited were from the hospital of the Ministry of National Guard Health Affairs (MNGHA), King Abdulaziz Medical City, Riyadh. Informed and written consent forms were obtained from all patients and healthy donors. Final recruitment for the study included 8 patients with AML, 8 patients with ALL, 8 patients with CML and 21 patients with CLL. In addition, 12 healthy donors (controls) were also recruited for comparative data analysis purposes (**Supplementary Table**).

### Cell Lines and Primary Leukemia Cell Isolation

Leukemic cell lines (THP-1, HL-60 and K562) were purchased commercially from ATCC (Manassas, VA) and a WA-C3CD5+ cell line was purchased from Leibniz Institute DSMZ - German Collection of Microorganisms and Cell Cultures. Cells were cultured in Roswell Park Memorial Institute (RPMI) media (Gibco) supplemented with 10% fetal bovine serum (Gibco, Life Technologies, Paisley, UK), 2mM L-glutamine (Gibco), 50U/ml penicillin and 50μg/ml in a 5% CO_2_ environment at 37°C. Peripheral blood samples and primary leukemic cells were collected from healthy donors or patients recently diagnosed with leukemia. Patient blood samples were obtained on the day of study enrolment and prior to receiving any chemotherapy. Peripheral blood mononuclear cells (PBMCs) were isolated from blood samples by centrifugation using Leucosep tubes (Greiner Bio-One) according to the manufacturer’s protocol. The isolation of the PBMCs was performed immediately after the blood sample was obtained. To avoid any effect of *in-vitro* cell culturing, the PBMCs were used for screening immediately after isolation.

### Drug Library and High Throughput Screening (HTS)

The library containing the 800 FDA approved drugs was provided by the Fraunhofer, Germany. All compounds were supplied at >90% purity as described by each vendor. All compounds were resuspended in dimethyl sulfoxide (DMSO) at a stock concentration of 10mM. For a single concentration screening (10µM final concentration), drugs were diluted to a concentration of 2mM and 0.1µl/well transferred using the ECHO acoustic dispenser (Labcyte, USA) into 384 well microtiter plates. Test compounds were dispensed into columns 3-22, media alone was dispensed into columns 1-2 (low control) and DMSO alone into columns 23-24 (0.5% v/v) which served as the high control. DMSO tolerance studies established that the cell lines were able to withstand 0.5% v/v DMSO for the purposes of the HTS campaigns. For the HTS campaigns, cells (PBMC or primary leukemic cells) were resuspended in complete RPMI and added to the 384 well microtiter plates at a density of 2,500 cells in 20μl media per well using the Multidrop Combi Reagent Dispenser (ThermoFisher, USA). For dose-response studies, 96 well microtiter plates were utilized using compound stock solutions (10mM) diluted in 100% v/v DMSO using a 1:3 serial dilution factor to yield 11-point dose-response curves. To perform the screening, 0.5µl of each diluted compound was dispensed (one compound/row) in wells 1-11 of each row and the last well contained 0.5μl DMSO (0.5% v/v) which served as the high control. For more detailed characterization of compounds, dose-response studies were performed using 16-point dose-response curves using a 1:2 serial dilution factor. Cells were added to each well of the microtiter plates at a density of 10,000 cells in 100μl media per well using the Multidrop liquid dispenser. In all HTS campaigns, cells were incubated with compounds for 48h at 37°C in the presence of 5% CO_2_ unless otherwise indicated. Cell viability was assessed using the CellTiter-Glo luminescent assay (Promega, UK) according to the manufacturer’s instructions. Briefly, an equivalent volume of CellTiter-Glo reagent (20µl for the 384 well assay and 100µl for the 96 well assay) was added to each well by the Multidrop liquid dispenser. After incubation in the dark for 15min at room temperature, the luminescence signal was measured using the EnVision plate reader (PerkinElmer, USA). Luminescence readings for cells exposed to compounds were normalized using the high and low control wells and the data were analyzed using GraphPad Prism (GraphPad Software Inc., USA) to yield the % cytotoxicity or IC_50_ values using a four parameter logistic regression model.

### Quantitative Reverse-Transcription-Polymerase Chain Reaction (qRT-PCR) Analysis of Adrenoceptor Beta Genes Expression

The expression of the three adrenoceptor beta genes ADRB1, ADRB2 and ADRB3 were assessed using the Taqman expression assays. Glyceraldehye-3-phosphate dehydrogenase (GAPDH) expression was used as internal control. All reactions were performed in a MicroAmp optical 384-well plate (Applied Biosystems) in a final volume of 5ul per reaction. For each reaction, 1ul of CDNA was used as the input for amplification. All primer-pairs used for the amplification of ADRB1, ADRB2 and ADRB3 and GAPDH were purchased from ThermoFisher and having the following catalogue numbers Hs00265096_s1,Human 79; Hs00240532_s1,HUMAN, 65; Hs00609046_m1, Human,65; Hs02758991_g1 respectively.

Amplification was performed as described in (15), briefly we used the 7900HT Fast Real-time PCR system (ThermoFisher) and the PCR cycling parameters were as follows: 95°C for 10mins followed by 40 cycles of PCR reactions at 95°C for 30 sec and 60°C for 1 min. The relative expression levels of the genes assayed in the cell synchronization validation method experiment were calculated using the comparative threshold cycle Ct (ΔΔCt) method. Initially, the Ct value of each gene was normalized to the corresponding Ct value of GAPDH for the same sample to obtain the relative threshold cycle (ΔCt). Following this the ΔCt for each biological replicate was then exponentially transformed into ΔCt expression by calculating 2 raised to the -ΔCt. Next, the average and standard deviation of the biological replicates were calculated followed by normalization to the average of the control samples for the same gene (ΔΔCt). Finally the ΔΔCt was calibrated from control samples and expressed as a relative fold-change. For relative mRNA analysis, raw Ct values were exported from ABI 7900 and imported into Microsoft Excel. Ct values were then used to calculate copy number for each well using historical/generic values from standard curves (Intersection at 0 = 40, slope = -3.5). Copy numbers were then normalized to GAPDH and mRNA levels were expressed relative to control samples.

### Western Blotting

All western blotting analysis were performed as previously described (16), Briefly, total protein extracts obtained by lysis of the PBMC or CLL primary cells (CPCs), were analyzed by the SDS-PAGE and transferred onto a polyvinylidene difluoride (PVDF) membrane. After a blocking step with 5% w/v bovine serum albumin (BSA) for 1h, PVDF membranes were incubated overnight at 4°C with different primary antibodies (Cell Signaling Technology, USA): anti-caspase-9 (1:1000, CST#9502S), anti-LC3 (1:1000 CST#4108S), anti-cleaved caspase-3 (1:1000, CST#9664S), anti-cleaved PARP (1:1000, CST#5625S) and anti-β-actin (1:3000, CST#3700S). The membranes were washed 3x (15min each) with 0.1% PBST and incubated with the corresponding HRP-conjugated secondary antibody for 1h at room temperature. Finally, after incubation of the membranes in a Clarity Western ECL Substrate Kit from Biorad, bands were visualized using the ChemiDoc MP imaging system (Biorad, USA) and the optical density (OD) was measured using ImageJ analysis software.

### Quantitation of Intracellular cAMP

To quantify intracellular cAMP concentrations, the cAMP-Screen Cyclic AMP Immunoassay System (ThermoFisher, USA) was utilized following the manufacturer’s protocol. Briefly, cell lysates from treated and non-treated cells were incubated with a cAMP-Alkaline phosphatase conjugate (cAMP-AP) and an anti-cAMP antibody in a coated microtiter plate. In the absence of intracellular cAMP, all cAMP-AP conjugate is captured on the coated surface, resulting in a high signal. When present in the cell lysate, intracellular cAMP competes with the cAMP-AP causing a reduced signal with signal reduction being proportional to the amount of cAMP present in the cell lysate. After washing to remove unbound cAMP-AP, the chemiluminescent alkaline phosphatase substrate is added, and the resulting glow signal is measured using a luminometer.

### AnnexinV/PI Staining

CPCs were treated with Isoprenaline for 48h then stained with Annexin-V/Propidium Iodide (PI) following the manufacture’s recommendations (BD Biosciences, USA) and analyzed using a FACSCanto II cytometer (BD Biosciences, USA).

### Combinatorial Treatment

During the combined treatment (Isoprenaline/fludarabine or Isoprenaline/Ibrutinib), cells were pre-treated for 12h with Isoprenaline to induce intracellular ATP depletion, after which fludarabine or Ibrutinib was added. Cells were re-incubated for 24h and cell cytotoxicity was assessed using propidium iodide staining.

### Data Analyses

The HTS screening data were analyzed using Activity Base (IDBS, Guildford, UK), and outlier elimination in the control wells was performed using the 3-sigma method. The raw data was normalized using the respective controls and the % effect of each compound in the assay calculated. Appropriate thresholds (% effect of each compound) were then used to select compounds for further progression, usually dose-response studies. For follow-up studies on individual compounds, Statistical significance of the experimental data was analyzed using the paired Student’s t-test. The observed differences between the experimental results were indicated as statistically significant if the *p*-value was smaller than 0·05 (*), 0·01 (**) or, 0·001 (***). To evaluate the synergism between Isoprenaline and fludarabine, a Co-operative Index (CI) was calculated as described (17) using the following formula: CI = (% Isoprenaline-induced cell death + % fludarabine-induced cell death)/% combined treatment-induced cell death. A Co-operative index lower than 1 indicates a synergistic effect.

## Results and Discussion

**Figure 1:**
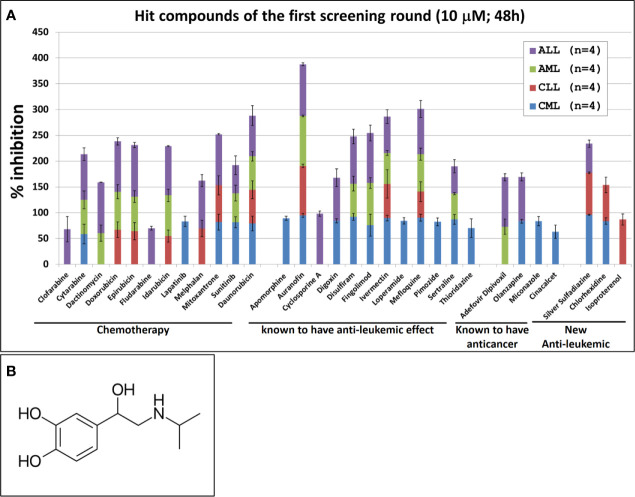
Hit compounds of the first round of screening. **(A)** List of Hit compounds selected in the phenotypic single-dose (10µM) HTS campaigns. Primary leukemic cells used in this screening were isolated from blood samples derived from patients with Acute Myeloid Leukemia (AML, n=4), Acute Lymphocytic Leukemia (ALL, n=4), Chronic Lymphoid Leukemia (CLL, n=4), Chronic Myeloid Leukemia (CML, n=4) and from four healthy donors (n=4). The overall screening results are displayed and expressed as mean of inhibition ± SD and normalized to DMSO (100%). **(B)** Chemical structure of Isoprenaline.

The single compound concentration HTS (10μM; 48h incubation) of the 800 compounds in the FDA approved drugs in primary cancer cells obtained from leukemic patients, led to the identification of 31 compounds that satisfied the criteria for progression (cytotoxic effect being at least 30% higher in leukemic cells than in normal PBMC). These compounds were subjected to 11-point dose-response studies to refine the observations in the HTS campaigns (**Figure 1A**). The majority of the selected hits (n=28) were known to have an anti-leukemic effect. The selection of these compounds provides evidence that the screening conditions were biologically relevant. Out of the 31 identified hits only three compounds showed an anti-leukemic effect for the first time: silver sulfadiazine, chlorhexidine and Isoprenaline. Silver sulfadiazine and chlorhexidine are approved for topical use only and are highly toxic if ingested (18, 19). Therefore, only Isoprenaline was selected for further pre-clinical characterization. Isoproterenol is a synthetic catechol compound (**Figure 1B**) and potent β-adrenergic agonist with peripheral vasodilator, bronchodilator, and cardiac stimulating properties. Isoproterenol exerts its effect on the β-1 adrenergic receptors in the myocardium, thereby increasing the heart rate and cardiac output. In addition, Isoproterenol acts on β-2 adrenergic receptors in bronchiolar and vascular smooth muscle, causing smooth muscle relaxation. Due to these properties, Isoprenaline is prescribed for the treatment of bradycardia, heart block, and rarely for asthma.

Pharmacologically, the effects of Isoprenaline are in part attributable to the stimulation of intracellular adenylyl cyclase through β-adrenergic receptors (20, 21). Due to Isoprenaline mediated stimulation, AC catalyzes the conversion of ATP to cyclic AMP (cAMP) and the increased intracellular cAMP levels are associated with relaxation of bronchial smooth muscle and inhibition of the release of mediators of immediate hypersensitivity from cells, especially from mast cells.

**Figure 2:**
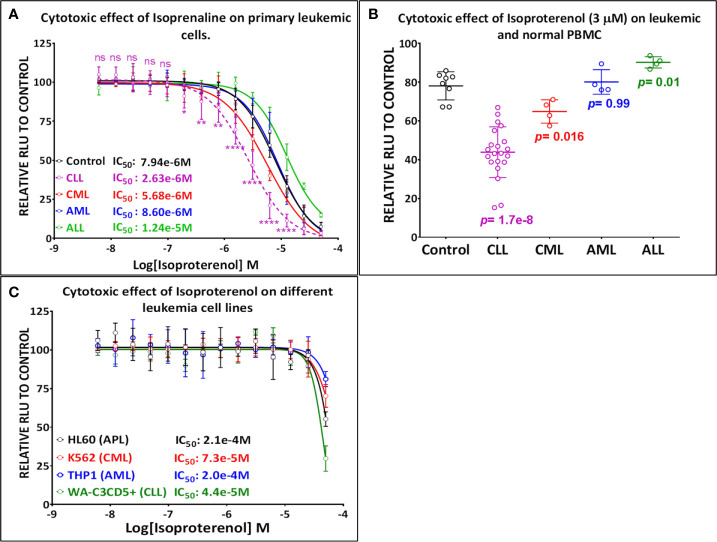
Primary leukemic cells deriving from CLL patients show the highest sensitivity to Isoprenaline. **(A)** Dose-response plots showing the cytotoxic effect of Isoprenaline on PBMC isolated from healthy donors (solid line) and leukemic patients (dashed line). Each dose-response curve represents the average of multiple samples testing; data are expressed as mean ± SD and normalized to DMSO (100%). Cell viability was determined by the CellTiter-Glo luminescence-based assay. CPCs are the only leukemic cells to show a significant higher sensitivity to Isoprenaline than the normal PBMCs. The p-values for each point (Isoprenaline concentration) of the plot were calculated with Student’s t test (ns, non-significant (P>0.05); *P < 0.05; **P < 0.01 and ****P < 0.0001). **(B)** The cytotoxic effect of a single dose (3µM) of Isoprenaline is compared for PBMC (n=8) and the four leukemia subtypes respectively [CLL (n= 23), CML (n=4), AML (n= 4), ALL (n=4)] (p values are indicated for each group). **(C)** Dose-dependent cytotoxic effect of Isoprenaline on different Leukemia cell lines.

Our screening results indicate that Isoprenaline has an anti-leukemic activity only on CLL primary cells (CPCs) (**Figure 1A**). To confirm this effect, fresh solid Isoprenaline was purchased and a dose-response testing was performed on all leukemia subtypes and normal PBMCs using the ATP-based luminescent CellTiter-Glo cell viability assay. Our results showed that Isoprenaline has cytotoxic activity on primary cancer cells derived from all leukemia subtypes AML, ALL, CML and CLL with IC_50_ of 8.6μM, 12.4μM, 5.6μM and 2.6μM respectively (**Figure 2A**). Only CPCs were more sensitive to Isoprenaline than the normal PBMCs. Isoprenaline showed no differential cytotoxicity between the primary cancer cells from other leukemia subtypes and the normal PBMCs (similar or higher IC_50_). Since one of the selection criteria for the screening was that the hit compound should have a specific cytotoxicity to leukemic cells, Isoprenaline was excluded from the hit list of single-dose screening in ALL, CML and AML because it did not fulfill this criterion (**Figure 1A**). The IC_50_ of Isoprenaline in CPCs is significantly lower (<3 times) than its counterpart in normal PBMCs (**Figure 2A**) suggesting an acceptable degree of specificity for CLL B-cells. A wide variability in the extent of the response to Isoprenaline treatment was observed in primary cancer cells isolated from different CLL patients (**Figure 2B** and **Supplementary Figure 1**), possibly due to status of the disease progression of the different donors (22, 23).

### Isoproterenol Induces Sustainable Dose-Dependent Intracellular ATP Depletion

**Figure 3:**
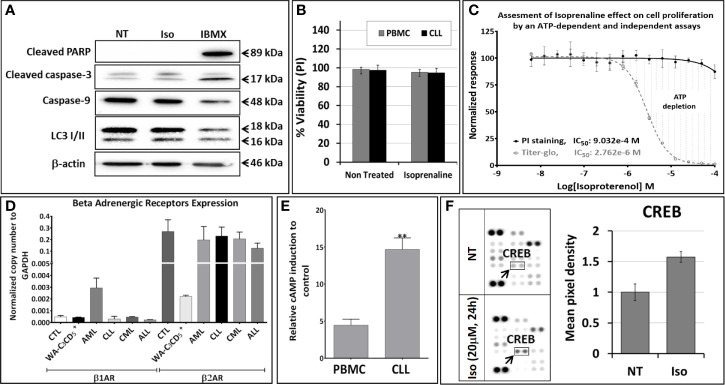
Isoproterenol does not induce cell death in primary CLL cells but it induces a long-lasting dose-dependent intracellular ATP depletion. **(A)** Primary CLL cells were treated with 3µM Isoprenaline (IC_50_ determined using the CellTiter-Glo assay) for 48h. Markers of apoptosis (cleaved PARP, cleaved caspase-3 and caspase-9) and autophagy (accumulation of LC3 II) were assessed by western blotting. The non-competitive selective phosphodiesterase inhibitor, IBMX, known to induce apoptosis in CLL B cells, was used as a positive control. Full-length blots/gels are presented in **Supplementary Figure 5**. **(B)** Isoprenaline-induced cell death in normal PBMC (black bars) and in primary CLL cells (gray bars) was also assessed by propidium iodide staining followed by flow cytometry analysis. **(C)** Cytotoxic effect induced by a gradient of Isoprenaline concentrations (dose-response plots) was assessed with an ATP-dependent (CellTiter-Glo, dashed line) and independent (PI/flow cytometry) assays. **(D)** The level of expression of the different adrenergic receptors in primary leukemic cells (ALL, AML, CLL and CML), normal PBMCs and in the CLL cell line WA-C3CD5+ was measured by quantitative real time PCR (qPCR). **(E)** Isoprenaline-induced intracellular cAMP accumulation in normal PBMCs and primary CLL cells pre-treated for one hour with phosphodiesterase inhibitor (IBMX) than with 10µM Isoprenaline for 24h. (**P < 0.01). **(F)** Human phosphokinase array reveals alteration in phosphorylation of kinases upon *in-vitro* treatment of CPCs with 10µM Isoprenaline for 24h. In the array each kinase is spotted in duplicate. Hybridization signals at the corners serve as control. Relative levels of protein phosphorylation (normalized intensity for each antibody) were calculated for each untreated and treated sample. p-CREB (indicated by black arrow) was the only significantly up-regulated kinase upon Isoprenaline treatment.

To determine the mechanism of action of Isoprenaline, we tested its effect on different leukemia cell lines, using the ATP-based CellTiter-Glo cell viability assay. Interestingly, all of these cell lines, including the CLL cell line WA-C3CD5+, showed a significant sensitivity to Isoprenaline cytotoxicity (**Figure 2C**). Since the cell lines were not reflecting what was seen in CPCs, the latter were used for all further characterization. It is noteworthy that after treating the CPCs for 48h with Isoprenaline at a concentration of 10μM, there was no difference in the cell-death markers (apoptosis and autophagy) between the treated cells and the control (**Figure 3A**). This observation was confirmed by microscopic examination after AnnexinV/PI staining (**Supplementary Figure 2A**). In addition, when we checked the cell viability with propidium iodide staining, no cell death was detected in the treated cells (**Figure 3B**). It is well established that Isoprenaline catalyzes the conversion of ATP to cAMP and thereby reducing the intracellular ATP concentration. Since we used an ATP-based cell viability assay (CellTiter-Glo) for the screening and subsequent testing, the Isoprenaline effect on the intracellular ATP may have interfered with its cytotoxic effect and the data generated by this assay could be misleading. To avoid such interference, we investigated the cytotoxic/anti-proliferative effect of Isoprenaline on the primary CLL cells using an ATP-independent cell viability assay, the cells were stained with propidium iodide (PI) and the cell viability was assessed by flow cytometry. As expected, this assay confirmed that the decrease in the luminescence signal was not induced by cell death but due to intracellular ATP depletion (**Figure 3C**). The PI-based cell viability assay indicated that the Isoprenaline-induced cell death was not observed at concentrations lower than 50μM for 48h. In terms of intracellular ATP depletion, the CPCs showed the highest sensitivity compared to normal PBMCs and the other leukemia subtypes (**Figure 2A**). To confirm the involvement of β-adrenergic receptors (ADRB) in this phenotype, we assessed their expression using real-time quantitative PCR (qPCR), in the CPCs, the normal PBMCs and in the CLL cell-line (WA-C3CD5+). We previously showed that Isoprenaline failed to induce ATP depletion in this cell line (**Figure 2C**). Our data indicated that all cells almost exclusively expressed the ADRB2, ADRB1 was expressed at very low levels and ADRB3 was undetectable in all samples (24). Since the difference of expression in ADRB2 dominated the difference in ADRB1, the total expression of the ADRBs was found to be almost 100 times higher in CPCs than in the Isoprenaline-resistant cell-line WA-C3CD5+ (**Figure 3D**). This data strongly suggest that Isoprenaline-induced intracellular ATP depletion is dependent on ADRB expression. If the data positively explained the different sensitivities of CPCs and the WA-C3CD5+ cell-line to Isoprenaline, it failed to explain the differences between the primary cancer cells isolated from the leukemia subtypes because all these cells have almost the same level of ADRBs expression (**Figure 3D**). This finding indicates that other pathways downstream of the ADRBs are involved in the Isoprenaline-induced ATP depletion. Intracellular ATP depletion is most likely the consequence of ATP conversion to cAMP by the adenylyl cyclase, activated by Isoprenaline through the β-adrenergic receptors (21). It is known that AC activity is higher in B lymphocytes than in other white blood cells (25) and because more than 80% of CPCs are B cells (25), the activity of AC in this leukemia subtype is expected to be higher. This explains why CLL had the highest sensitivity to Isoprenaline in terms of intracellular ATP depletion compared to the other leukemia subtypes and the normal PBMCs.

Isoprenaline-induced intracellular ATP depletion in CPCs begins as early as one hour after Isoprenaline treatment (**Supplementary Figure 2B**). Isoprenaline treatment induces an accumulation of intracellular cAMP in normal PBMCs and in CPCs. The intracellular cAMP accumulation was significantly higher in CPCs (**Figure 3E**). Isoprenaline-induced cAMP accumulation was also confirmed by the phosphorylation of the cAMP-response element binding protein (CREB) in Isoprenaline treated CPCs (**Figure 3F**). The relatively higher accumulation of cAMP is an additional indication that the AC activity in primary CLL cells is higher than in normal PBMCs. The difference in intracellular cAMP accumulation is most probably related to the higher activity and/or expression of ADRB and AC in CLL B-Cells (25).

**Figure 4:**
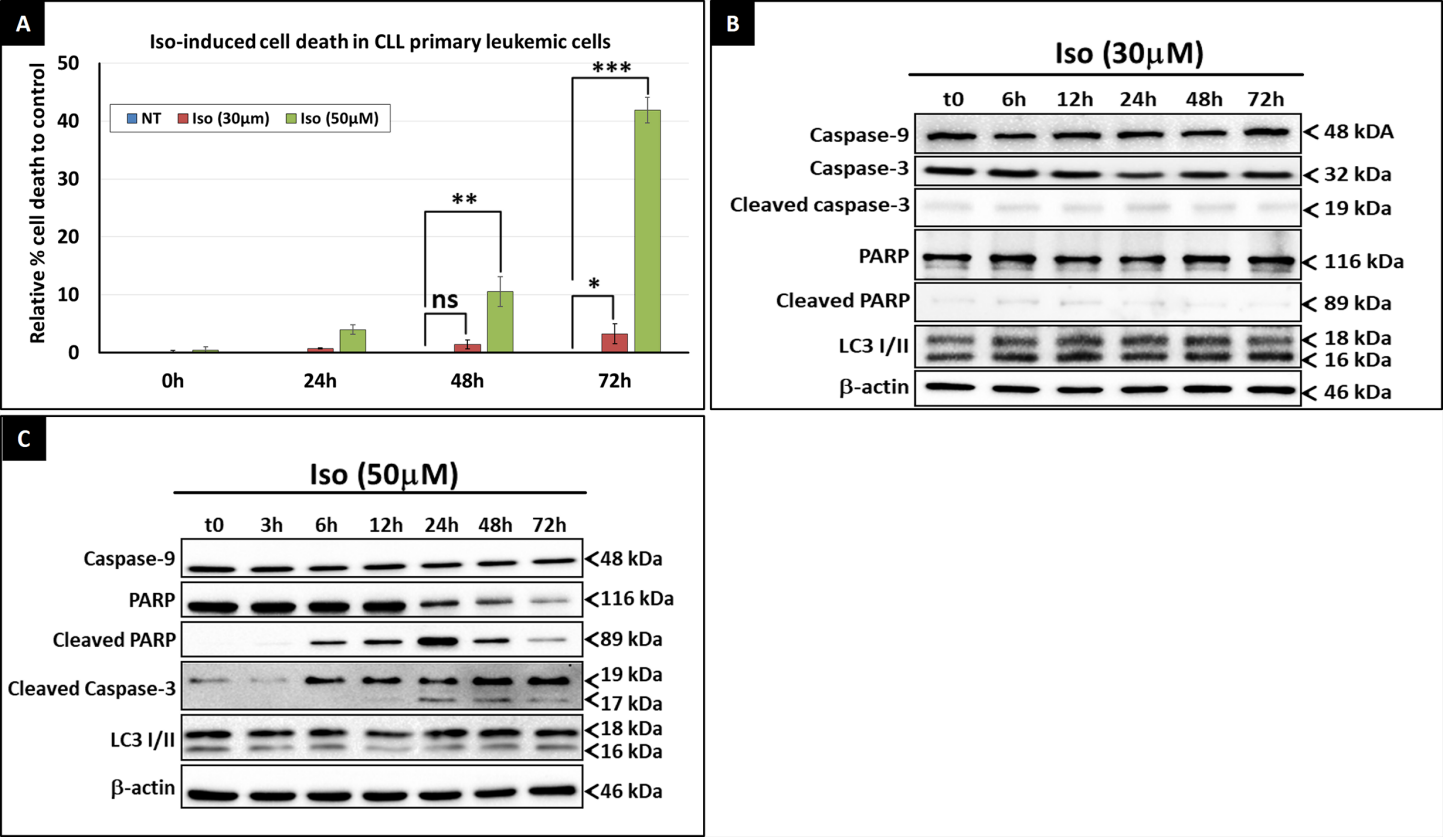
Isoprenaline cytotoxic effect in CLL primary cells. **(A)** Cell viability assessment after the treatment of CPCs with either low dose of Isoprenaline (red bars) or high dose (green bars). The graph shows the relative cell-death rate to the control (basic spontaneous cell-death percentage observed in the control was subtracted from the rate observed in treated cells). Time course assessment of the cytotoxic effect of, low **(B)** and high **(C)** doses of Isoprenaline, through the quantification of apoptotic (PARP, caspase-9 and -3) and autophagic (accumulation of LC3 II) markers. [ns, non-significant (P > 0.05); *P < 0.05; **P < 0.01 and ***P < 0.001)]. Full-length blots/gels used to generate panels **(B)** and **(C)** are presented in **Supplementary Figures 6** and **7** respectively.

Despite its significant effect on the intracellular ATP level, Isoprenaline failed to induce cell death in CPCs at concentrations lower than 50μM (**Figure 3C**). Beyond this concentration, intracellular ATP is reduced to less than 15% of the normal level, which is too low to sustain cell viability and cell death occurred. This observation is in concordance with the results of previous studies conducted by Nieminen et al. and Sweet et al. showing that an intracellular ATP reduction above 15%, does not induce cell-death, but arrests proliferation (26, 27). To confirm the nature of Isoprenaline-induced cell death, CPCs were incubated with Isoprenaline (50μM) for 48h. The cells were then double stained with propidium iodide (cell death marker) and AnnexinV (apoptotic marker). The flow cytometry analysis of the treated cells indicated that, even at such a high concentration, Isoprenaline induced cell-death only in a very small population of CPCs (**Figure 4A**). At a concentration lower than 30μM, a longer incubation period (72h) was required to observe cell death (**Figure 4A**), but apoptotic and autophagic markers were similar to the control even after long-term treatment (**Figure 4B**). It is well established that the intracellular ATP level is a key element in determining the cell death fate (26). Intracellular ATP is necessary for the conversion of pro-caspase-9 to activated caspase-9 and the subsequent induction of apoptosis (28, 29). A low level of intracellular ATP completely blocks Fas/Apo-1-stimulated apoptosis and cells undergo a necrotic cell death process (30). Our data, combined with the results of the above-mentioned studies, indicates that Isoprenaline (30μM) induces a drastic intracellular ATP depletion leading at long term to necrotic cell death in primary CLL cells (26, 30, 31).

As previously demonstrated by the MTT cell-viability assay, Isoprenaline-induced cell-death was observed only at concentrations higher than 50μM (**Figure 3C**). Long-term treatment of primary CLL cells with 50μM Isoprenaline showed a specific increasing cell-death rate at 48 and 72h incubation (**Figure 4A**). Western blotting analysis of apoptotic (caspase-3, -9 and cleaved PARP) and autophagic (accumulation of LC3II) markers showed that Isoprenaline induced very early (6h) PARP and caspase-3 cleavage. This indicates that cells were undergoing an apoptotic cell-death. However, caspase-9 remained unchanged. This result confirmed that intracellular ATP is needed for the activation of pro-caspase-9 (28, 29) and indicates that Isoprenaline induced caspase-9-independent apoptotic cell death in CPCs (**Figure 4C**) (32, 33).

### Isoprenaline Combination With the Purine Analogue Fludarabine

Acquired chemoresistance is a major impediment to successful chemotherapy. To adapt to genotoxic stress and to escape the cytotoxicity of drugs, cancer cells acquire different genetic and epigenetic alterations. The majority of these pro-survival alterations are ATP-dependent, such as drug efflux, DNA damage repair and activation of intracellular survival signaling (34). The intracellular ATP level is a core determinant in the development of chemoresistance in many cancer cell lines (35, 36). Zhou et al. showed that treatment of cross-resistant cells with the glycolysis inhibitor, 3-bromopyruvate, decreased their intracellular ATP level sufficiently to sensitize them to multiple chemotherapeutic drugs (35).

Since the discovery of their clinical efficacy in CLL treatment, purine-analogues became the chosen first line of therapy, being preferred above alkylating agents, as a central component in modern therapy (37). Despite the promising chemotherapeutic efficacy of purine analogues, resistance to these drugs remains a major clinical problem, which requires a thorough understanding of drug action at the cellular level to solve. In addition to its role in chemoresistance, intracellular ATP constitutes a specific limiting factor in purine analogue-based chemotherapy. Intracellular ATP and purine analogues compete for the same target enzymes (DNA polymerases, DNA primase, DNA ligase I) (38). The higher the concentration of intracellular ATP, the lower the binding of purine analogues to their targets causing limited efficacy and ultimately resistance. We hypothesized that decreasing the intracellular ATP content will reduce the competition with purine analogues, improving their efficacy.

**Figure 5:**
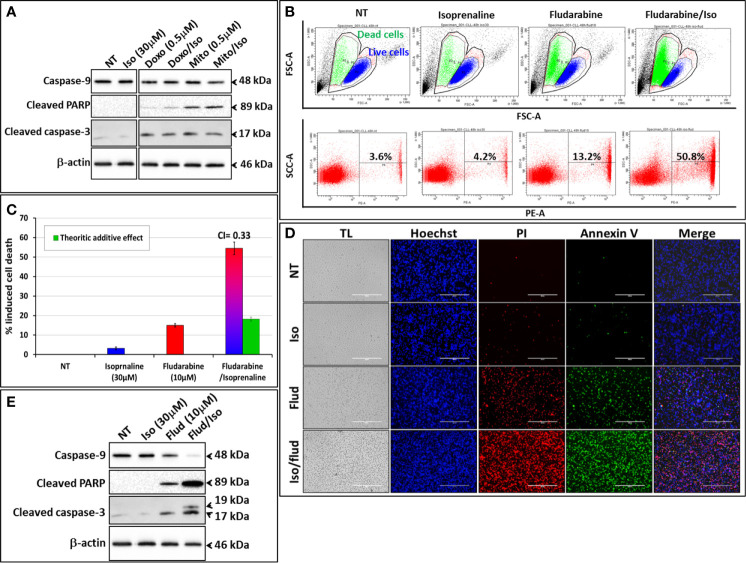
Isoprenaline potentiates fludarabine effect to synergistically induce apoptotic cell death in CLL primary cells. CPCs were pre-treated with Isoprenaline (30μM) for 12h, to allow intracellular ATP depletion, prior to the addition of the second drug of the combination. **(A)** Effect of the combinatorial treatment of Isoprenaline with different anthracyclines (Doxorubicin and Mitoxantrone) on CPCs. Cytotoxic effects of the different treatment were assessed through the quantification of apoptotic cell death markers (caspase-3, -9 and cleaved PARP). All cut and reassembled bands belong to the same blot-images (see **Supplementary Figure 8** for original blot images). **(B)** Cytotoxicity/cell-growth inhibition of primary CLL cells by the purine analogue fludarabine (10μM) alone or in combination with Isoprenaline (30µM). Induced cell death was assessed by PI staining followed by flow cytometry analysis. **(C)** Average cytotoxic effect of Isoprenaline/Fludarabine combination on leukemic cells isolated from 5 CLL patients. The Cooperation Index **(CI)** between Isoprenaline and fludarabine was calculated, and was far below 1 indicating a high synergism between these two drugs. **(D)** Fluorescent microscopic images showing CPCs treated with fludarabine (10μM) alone or in combination with Isoprenaline (30μM). The cells were stained with Hoechst (blue), AnnexinV (green), PI (red). **(E)** Assessment of Isoprenaline/fludarabine combination on apoptotic cell-death markers (caspase-3, -9 and PARP) in CPCs. Full-length blots/gels are presented in **Supplementary Figure 8**.

We exploited the ability of Isoprenaline to deplete intracellular ATP in CPCs to assess its ability in sensitizing the cells to the purine-analogue (fludarabine). Combinatorial treatment of CPCs with Isoprenaline and different anthracyclines (Doxorubicin and Mitoxantrone) showed additive cytotoxic effects (**Figure 5A**), but when combined with the purine-analogue (fludarabine), Isoprenaline treatment showed a high synergistic effect. This synergism was assessed with AnnexinV/PI staining followed by either flow-cytometry (**Figures 5B, C**) or microscopy (**Figure 5D**). The observed synergism of Isoprenaline and fludarabine was also confirmed by the quantification of apoptotic-death markers (**Figure 5E**). Pre-treatment of the CPCs with 30μM Isoprenaline for 12h prior to fludarabine (10μM) addition induces a cell-death rate of 54.5%, which is almost 3 times higher than the theoretic additive effect (18.2%) (**Figure 5C**). To determine whether the combinatorial effect was dependent on the sequence of the treatment, we evaluated the sequence of the addition in this two-drug combination. The results showed that, when Isoprenaline is added simultaneously or after fludarabine treatment, the synergistic effect decreases dramatically (data not shown). This result is expected, because a pre-treatment with Isoprenaline will induce intracellular ATP depletion, the main competitor of fludarabine, leading to a potentiation of fludarabine’s efficacy.

**Figure 6:**
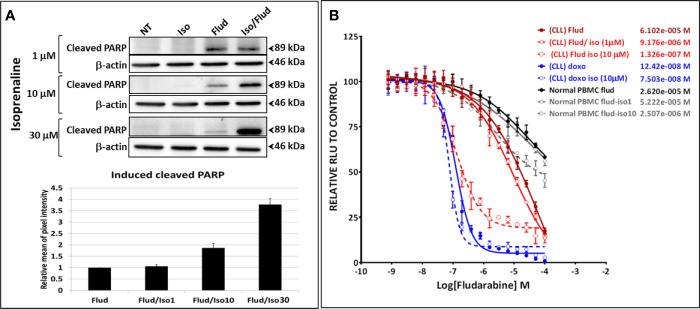
Isoprenaline synergizes specifically with the purine analogue fludarabine in dose-dependent manner. **(A)** The cytotoxic effect of fludarabine (10μM) combined with an increasing concentration of Isoprenaline (1, 10, 30μM) was assessed through the quantification of the apoptotic marker (cleaved PARP). Full-length blots/gels are presented in **Supplementary Figure 9**. **(B)** CPCs were treated with a gradient of fludarabine (red plots) or Doxorubicin (blue plots) concentrations combined with either 1μM or 10μM Isoprenaline. The cytotoxic effect of Fludarabine/Isoprenaline combination was also assessed in PBMC isolated from normal donors (gray scale plots).

The combinatorial treatment of leukemic primary cells, isolated from patients with lower sensitivity to Isoprenaline (CLL with lower sensitivity, normal donors or donors with other leukemia subtypes) showed lower synergistic effects (**Supplementary Figure 3**). These results indicate that the observed synergism is proportional to Isoprenaline-sensitivity, in other words proportional to the efficacy of Isoprenaline in depleting the intracellular ATP. This observation was confirmed by the combination of different doses of Isoprenaline (0, 1, 10 and 30μM) with a constant concentration of fludarabine (10μM) (**Figures 6A, B**). The results indicated that none of the doses of Isoprenaline induced any cell death but when combined with fludarabine, a proportional synergistic effect was observed (**Figure 6A**). We also tested the effect of Isoprenaline on fludarabine efficiency (IC_50_) in killing CPCs. Different doses of Isoprenaline (0, 1 and 10μM) were combined with a gradient concentration of fludarabine and doxorubicin (dose-response). As shown previously, the Isoprenaline combination with doxorubicin (anthracycline) produced a slightly decreased IC_50_ with a minor shift of the dose-response curve to the left (**Figure 6B**, blue plots). However, when combined with fludarabine, Isoprenaline induced a drastic decrease of fludarabine IC_50_ and a major left-shift of the drug-response plot (**Figure 6B**, red plots). In primary CLL cells, 1 μM and 10 μM doses of Isoprenaline induced a reduction of fludarabine IC_50_ by 6 and 470-fold respectively. The same combinations were tested on PBMCs isolated from healthy donors (**Figure 6B**, gray-scale plots). The results showed that Isoprenaline and fludarabine still synergized but with to a relatively minor extent. A 10µM dose of Isoprenaline induced a reduction of the fludarabine IC_50_ only 10-fold. In combination, these results provide evidence that the Isoprenaline/fludarabine combination selectively targets CPCs.

To evaluate the synergism between Isoprenaline and fludarabine, we also calculated the Co-operative Index (CI) between these two drugs (**Supplementary Figure 4A**). We evaluated also the effect of each drug on their CI. Results indicated that a combination of fludarabine with increasing doses of Isoprenaline induces a decrease of CI (higher synergism) (**Supplementary Figure 4B**), however fludarabine concentration did not show any significant effect on CI (**Supplementary Figure 4C**). This data confirmed that Isoprenaline concentration is the driving factor of the observed synergy.

**Figure 7:**
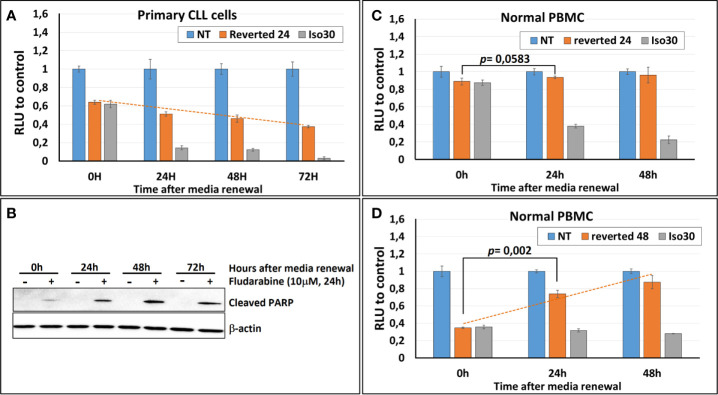
Isoprenaline-effect on intracellular ATP content is irreversible in CLL primary cells. **(A)** Isoprenaline induced a long-lasting intracellular ATP depletion. The concentration of intracellular ATP in CLL primary cells was measured over 72 h in: i) non-treated cells (blue bars), ii) cells treated with Isoprenaline for only 24h then Isoprenaline was removed by cell wash and cells were maintained in Isoprenaline-free media (orange bars), or iii) in cells permanently treated with Isoprenaline (gray bars). Concentrations of intracellular ATP at the different time points were normalized to the respective negative control (non-treated cells). **(B)** Primary CLL cells were treated with Isoprenaline for only 24h then Isoprenaline was removed by cell wash and cells were maintained in Isoprenaline-free media, fludarabine (10μM) was added at different time points after Isoprenaline removal (0, 24, 48 and 72h). Primary CLL cells were incubated with fludarabine for 24h then fludarabine cytotoxic effect was assessed through quantification of the apoptotic marker (cleaved PARP). Full-length blots/gels are presented in **Supplementary Figure 10**. **(C)** Normal PBMC were treated as in **(A)**. **(D)** Normal PBMC were treated as in **(A)** except that media was renewed after 48 hours instead of 24 hours.

To assess the reversibility of the Isoprenaline effect on intracellular ATP, CPCs were incubated for 24h with 30μM Isoprenaline, extensively washed and re-incubated with normal media (no Isoprenaline). The intracellular ATP in these cells was quantified using the ATP-based assay CellTiter-Glo at different time points to determine the speed of recovery. The content of the intracellular ATP was assessed at different time points and compared to non-treated and permanently treated cells. The results showed that Isoprenaline decreases intracellular ATP in a time-dependent manner (**Figure 7A**, gray bars) and induced irreversible intracellular ATP depletion, as the cells failed to recover their initial intracellular ATP concentration after drug alleviation (**Figure 7A**, orange bars). To further demonstrate the Isoprenaline-induced irreversible ATP depletion, CPCs were treated with Isoprenaline (30μM) for 24 h then culture media was removed and replaced with Isoprenaline-free media. Fludarabine (10μM) was added to the treated CPCs at different time points post Isoprenaline removal. After an incubation of 24h, fludarabine-induced cytotoxicity was assessed through the quantification of the apoptotic marker (cleaved PARP). Results showed that, the more delayed the addition of fludarabine the higher its cytotoxic effect (**Figure 7B**). The increased cytotoxicity of fludarabine is indicative of an increased intracellular ATP depletion.

Isoprenaline-induced irreversible ATP depletion is suggestive of permanent mitochondrial damage and impaired ATP production. This hypothesis is supported by the results of previous studies showing that Isoprenaline induces mitochondrial disruption in the cardiomyocyte cell-line H9C2 (39, 40).

To investigate the reversibility of Isoprenaline-induced ATP depletion in normal PBMCs these cells were treated in the same manner as described above (30μM Isoprenaline for 24h). The amount of intracellular ATP depleted due to this treatment was very low and difference between treated and control PBMC was negligible (**Figure 7C**, orange bars). This result is due to the relatively low sensitivity of normal PBMC to Isoprenaline by comparison to primary CLL cells. This difference in sensitivity constitutes a comfortable therapeutic window if this combination (Isoprenaline/Fludarabine) is considered for treatment of CLL.

To make the testing of the reversibility possible and to induce a significant intracellular ATP depletion, normal PBMC were incubated with Isoprenaline (30μM) for a longer period (48h), before Isoprenaline alleviation. Intracellular ATP assessment at different time-points showed a significant increase of intracellular ATP in normal PBMC treated with Isoprenaline after media renewal (**Figure 7D**, dashed orange line). This result indicates that, contrary to primary CLL cells that failed in recovering the physiological amount of intracellular ATP after Isoprenaline treatment, normal PBMC were able to revert Isoprenaline effect and reconstitute their stock of intracellular ATP.

This difference in the ability of reverting Isoprenaline-induced intracellular ATP depletion between leukemic cells and normal PBMC is a good indication that Isoprenaline has no permanent damage on normal PBMC and subsequently it could constitute a safe therapeutic option for CLL treatment. Though the molecular mechanism involved in intracellular ATP depletion reversibility/irreversibility has to be investigated.

### Isoprenaline Combination With the Bruton’s Tyrosine Kinase Inhibitor Ibrutinib

Until recently, systemic chemoimmunotherapy, based on fludarabine/cyclophosphamide/rituximab (FCR) and bendamustine/rituximab (BR) regimens has been considered the standard of care for frontline management in CLL treatment. These regimens demonstrated excellent response rates, progression-free survival and overall survival in the patients who could tolerate them. Nonetheless, significant myelosuppression and infectious complications made the use of these regimens difficult to tolerate in elderly patients with comorbidities. Moreover, these regimens showed lower efficiency in patients with 17p (del17p) deletion or TP53 mutation due to a high rate of relapse shortly after chemoimmunotherapy. The approval of the first-in-class Bruton’s tyrosine kinase inhibitor (BTKi), ibrutinib was a revolution in the treatment of del17p/TP53mut patients, achieving an overall survival never seen with prior therapies. Shortly after, ibrutinib was approved for all patients and the frontline treatment paradigm has been changed. BTKi are now preferred regimens for the initial treatment of CLL, with or without del17p due to its marked efficacy and tolerability in treatment-naïve, relapsed/refractory chronic lymphocytic leukemia/small lymphocytic lymphoma and mantle cell lymphoma. Ibrutinib has also been used as a novel anticancer drug for several solid tumors such as breast, ovarian, gastric and lung cancer.

Ibrutinib is a small-molecule covalent inhibitor of BTK, which selectively and irreversibly inhibits pBTK. Ibrutinib binding site overlaps with the ATP binding pocket on BTK preventing ATP from binding, thereby preventing Btk phosphorylation and activation.

**Figure 8:**
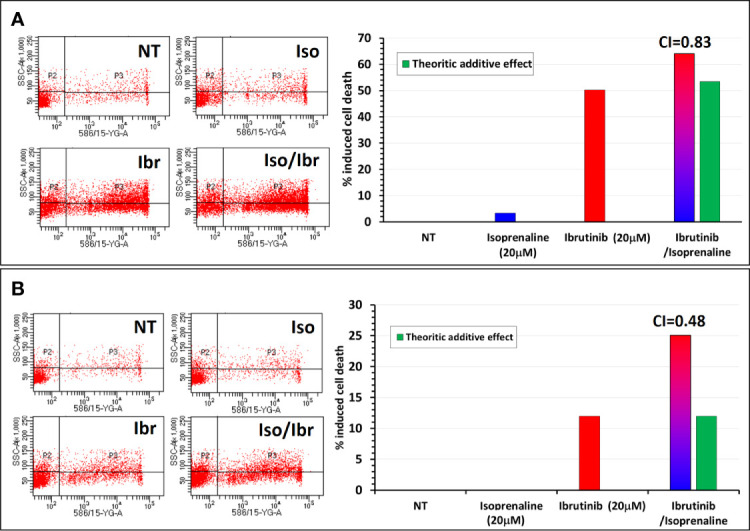
Synergistic effect between Isoprenaline and the Bruton’s tyrosine kinase inhibitor (Ibrutinib). **(A)** Combinatorial treatment of CLL primary cancer cells with Isoprenaline (20μM) and Ibrutinib (20 μM) shows that Isoprenaline sensitizes CLL primary cancer cells to the cytotoxic effect of Ibrutinib. **(B)** This synergistic effect between Isoprenaline and Ibrutinib was more pronounced (CI=0.48) in CPCs showing higher resistance to Ibrutinib than in more sensitive CPCs (CI=0.83).

Since ibrutinib and ATP are competing for the same binding site on BTK, a depletion of intracellular ATP should theoretically facilitate ibrutinib binding to BTK and subsequently increase its potency like what was observed with fludarabine. To verify this hypothesis, CPCs freshly isolated were treated *in vitro* with either Ibrutinib, Isoprenaline or a combination of these two drugs. Combinatorial treatment showed a synergistic effect between Isoprenaline and ibrutinib. This synergism was assessed with PI staining followed by flow-cytometry (**Figure 8**). Pre-treatment of the CPCs with 20μM Isoprenaline for 12h (to allow intracellular ATP depletion) prior to ibrutinib (20μM) addition induces a cell-death rate (red/blue gradient bars) higher than the theoretic additive effect (green bars). This synergism between Isoprenaline and ibrutinib is more pronounced in CPCs with higher resistance to ibrutinib (**Figure 8B**). Despite the promising therapeutic efficacy of ibrutinib in CLL, relapse and acquired resistance to ibrutinib are still observed, consequently these kind of combinatorial therapies may improve the rate of complete remission and overcome resistance to ibrutinib.

## Conclusion

There is a growing interest in drug combinations for cancer therapy and prevention (41–43). There is also an increasing awareness that the use of synergistic drug combinations allows for lower doses of each drug-constituent with lower adverse effects without jeopardizing therapeutic efficiency. We showed in this study by phenotypic screening that treatment of CPCs with the β-adrenergic agonist Isoprenaline sensitizes the CPCs to the purine analogue chemotherapeutic drug fludarabine. Combinatorial treatment with these two drugs induced a synergistic enhanced tumor-cell killing selectively in CPCs. To evaluate its clinical efficacy, toxicity level and prevention of rapid onset of resistance, this combination has to be tested in a xenograft or CLL mouse model and ultimately in clinical trials. In theory, intracellular ATP constitutes the main competitor for purine analogues and derivatives used in CLL treatment. Intracellular ATP depletion following Isoprenaline treatment should potentiate their toxicity. This synergistic effect was confirmed for Fludarabine but when Isoprenaline was combined with other purine analogues such as Mercaptopurine or with drugs having Purine analog-like properties, such as Bendamustine, no synergistic effect was observed (data not shown). Targeted therapies based on BTK inhibitors, such as Ibrutinib, become frontline treatment for CLL patients. We showed that depletion of intracellular ATP by Isoprenaline facilitated the binding of this inhibitor and subsequently increased its potency leading to a synergistic effect between Isoprenaline and Ibrutinib. This synergism was more pronounced in CLL primary cancer cells with lower sensitivity to Ibrutinib.

Due to the wide, almost ubiquitous expression of ADRBs (44, 45), Isoprenaline-based therapies have many adverse effects (46, 47). To limit its toxicity and spread in the body, Isoprenaline could be sequestered through conjugation to a CLL B-cell specific antibody such as alemtuzumab, obinutuzumab, ofatumumab or rituximab in the circulation system. An Isoprenaline-antibody conjugate and fludarabine combination has to be tested *in-vitro* and *in-vivo* to verify if the synergistic effect is preserved after conjugation. FCR (fludarabine, cyclophosphamide, and rituximab) is the current standard treatment for CLL, if the Isoprenaline-rituximab conjugate show a promising synergistic effect with fludarabine, this ADC could replace rituximab in the treatment-regimen, allowing a reduction of the administrated dose of fludarabine and consequently the associated adverse effects.

Anti-folates, a class of anti-metabolite medications that block purine *de-novo* biosynthesis, constitute a potential candidate for Isoprenaline substitution in this combination due to their ability to deplete intracellular ATP (48–51). Different anti-folates such as Methotrexate and Pemetrexed are approved as an anti-neoplastic agent for the treatment of hematologic malignancies and non-small cell lung carcinoma. A combination of these anti-folates with the purine analogue fludarabine should be investigated for a possible synergistic effect.

## Data Availability Statement

The original contributions presented in the study are included in the article/**Supplementary Material**. Further inquiries can be directed to the corresponding authors.

## Ethics Statement

The study protocol was approved by the Institutional Review Board of King Abdullah International Medical Research Center and all methods were performed in accordance with the World Medical association declaration of Helsinki on ethical principles for medical research involving human subjects. Informed and written consent forms were obtained from all patients and healthy donors.

## Author Contributions

AN and MB conceived and designed the project. AN and NS performed all drug toxicity, drug combination, cell culture and staining, flow cytometry experiments, analyzed the data and wrote the manuscript. AN and AAlh performed the high throughput drug screening. AAlh performed qPCR experiments. AN, SG, and JR analyzed the high throughput screening data. AN and AM performed western blotting experiments. AAlh, TT, AAla, GG, and KA recruited patients and helped in sample collection. FM helped in cell culture. All authors contributed to the article and approved the submitted version.

## Funding

We declare that all sources of funding received for this research are submitted. This work was supported by King Abdullah International Medical Research Center (KAIMRC) Institutional Research Grant (grant number RC17/006/R) to MB and AN.

## Conflict of Interest

The authors declare that the research was conducted in the absence of any commercial or financial relationships that could be construed as a potential conflict of interest.
