# Supplementary material for: A Drug Repositioning Approach Identifies a Combination of Compounds as a Potential Regimen for Chronic Lymphocytic Leukemia Treatment
Source: Front Oncol. 2021 May 28;11:579488. doi: 10.3389/fonc.2021.579488 (PMC8195271; doi:10.3389/fonc.2021.579488)
Supplement: Supplementary file 1 [file DataSheet_1.pdf]

A drug repositioning approach identifies a combination of compounds as a potential regimen for Chronic Lymphocytic Leukemia treatment

Atef Nehdi; Nosaibah Samman; Abdullah Mashhour; Alshaimaa Alhallaj; Thadeo Trivilegio; Sheraz Gul; Jeanette Reinshagen; Ahmed Alaskar; Gamal Gmati; Khadega A Abuelgasim; Fatmah Mansour and Mohamed Boudjelal.

Supplementary Figures

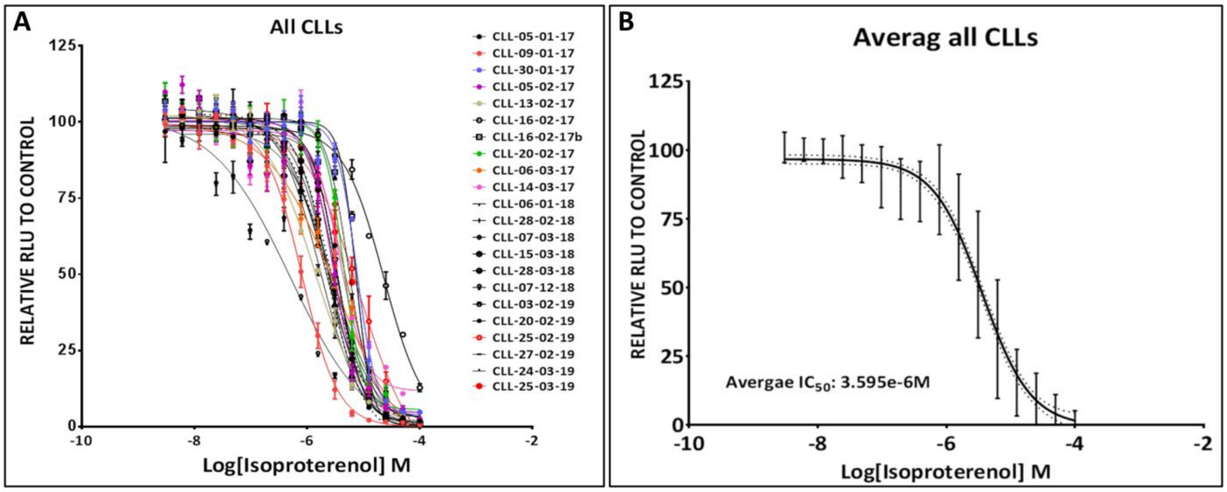

Supplementary Figure 1: Variability of patient sensitivity to Isoprenaline. **(A)** Dose-response curves showing the cytotoxic effect of Isoprenaline on primary CLL cells isolated from 22 patients. **(B)** Average of all dose-response plots represented in (A). The size of error bars indicates the variability in patient sensitivity to Isoprenaline.

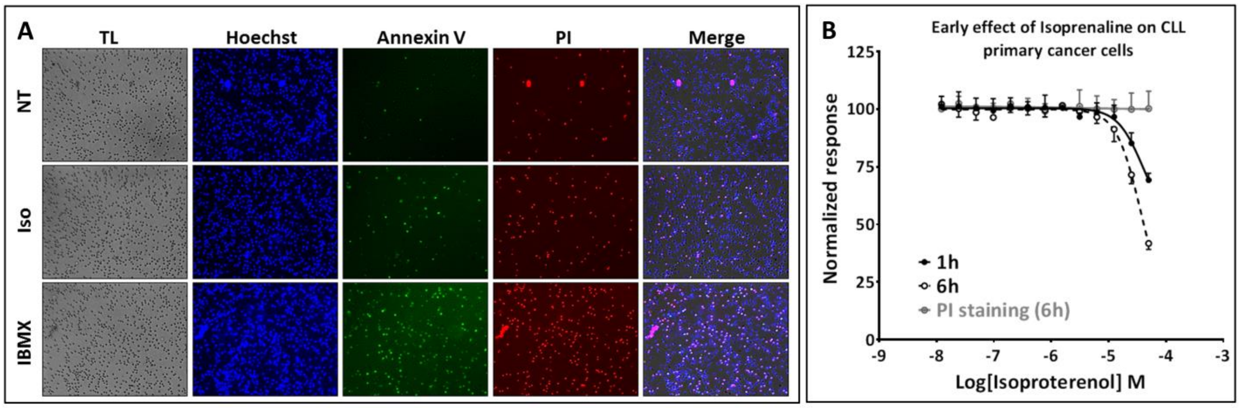

Supplementary figure 2: Isoprenaline failed to induce cell death in primary cells but it induced intracellular ATP depletion that started as early as one hour after Isoprenaline treatment. (A) CLL primary cells were incubated 48h with Isoprenaline (10μM). After AnnexinV/PI staining cells were examined by fluorescent microscopy. B) Primary CLL cells were treated with a serial dilution of Isoprenaline. Dose-dependent intracellular ATP depletion was followed by the ATP-based assay CellTiter-Glo at different time points (1h and 6 h after treatment). Cell viability was assessed by PI staining followed by flow cytometry analysis.

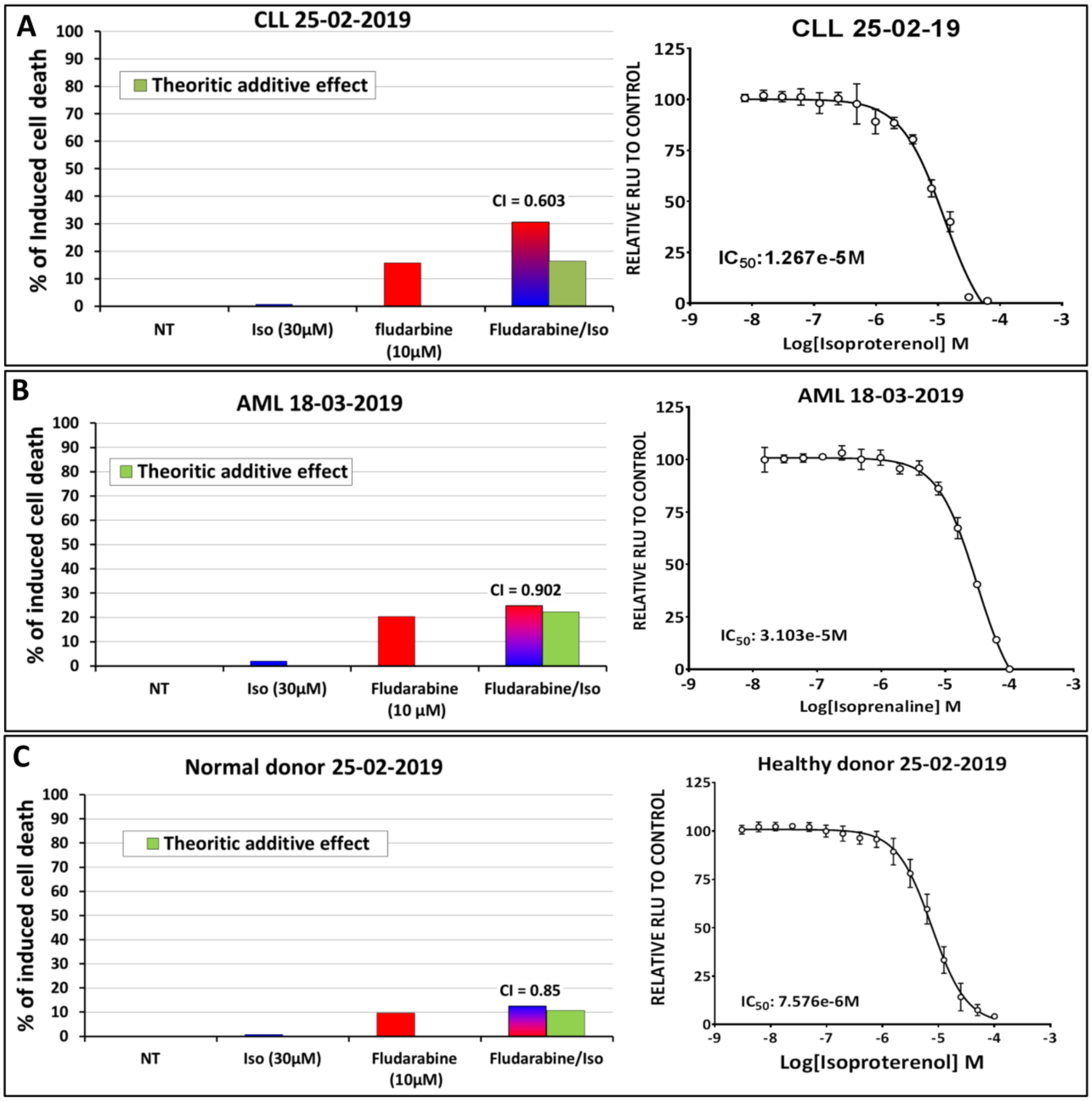

Supplementary Figure 3: Synergistic effect between fludarabine and Isoprenaline is proportional to the sensitivity of treated cells to Isoprenaline. The efficiency of Isoprenaline in depleting intracellular ATP is represented by the dose-response curves (IC_{50}). Isoprenaline induced intercellular ATP depletion was determined by the ATP-based luminescent CellTiter-Glo assay. The indicated IC_{50} values are inversely proportional to the efficiency of Isoprenaline in depleting intracellular ATP. Leukemic Primary cells isolated from a CLL patient at early stage **(A)**, and an AML Patient **(B)** and normal PBMC isolated from a normal donor **(C)** showed lesser sensitivity to Isoprenaline. As indicated by the different CI values, combinatorial treatment of these samples showed relatively low synergism between Isoprenaline and fludarabine.

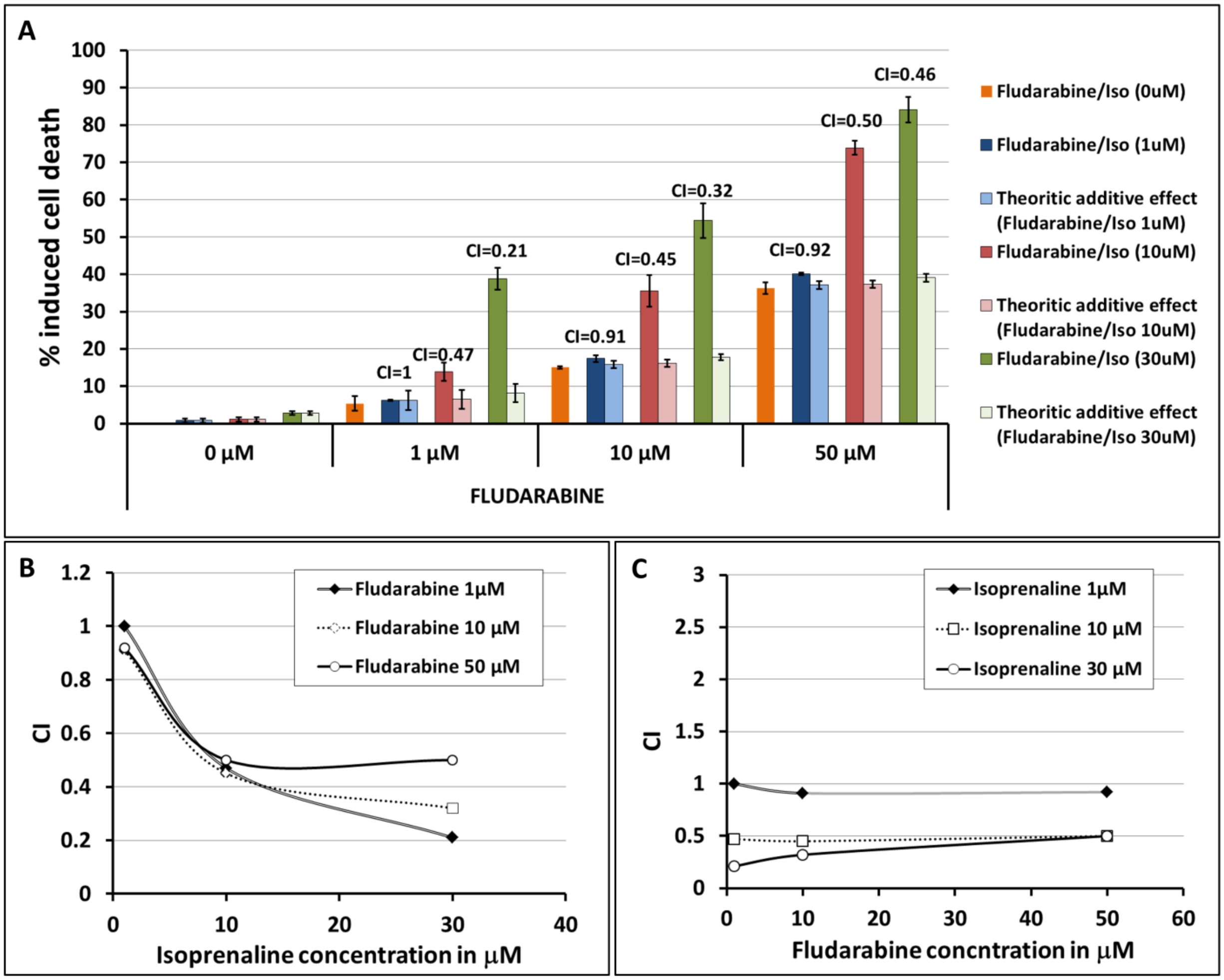

Supplementary Figure 4: Isoprenaline concentration is the driving factor of its synergism with fludarabine. We tested the effect of Isoprenaline and fludarabine concentration on their synergistic cytotoxicity in primary CLL cells. **(A)** CLL primary cells were treated with different concentrations of Isoprenaline (0 (orange bars), 1 (dark blue bars), 10 (red bars) and 30µM (green bars)) combined with different concentration of fludarabine (0, 1, 10 and 50µM). The theoretic additive effect and the Cooperation Index (**CI**) for each combination were calculated and represented. The effects of Isoprenaline **(B)** and fludarabine **(C)** concentrations on their synergistic cytotoxic effect (cooperation index CI) were evaluated.

Blots used to generate figure 3A

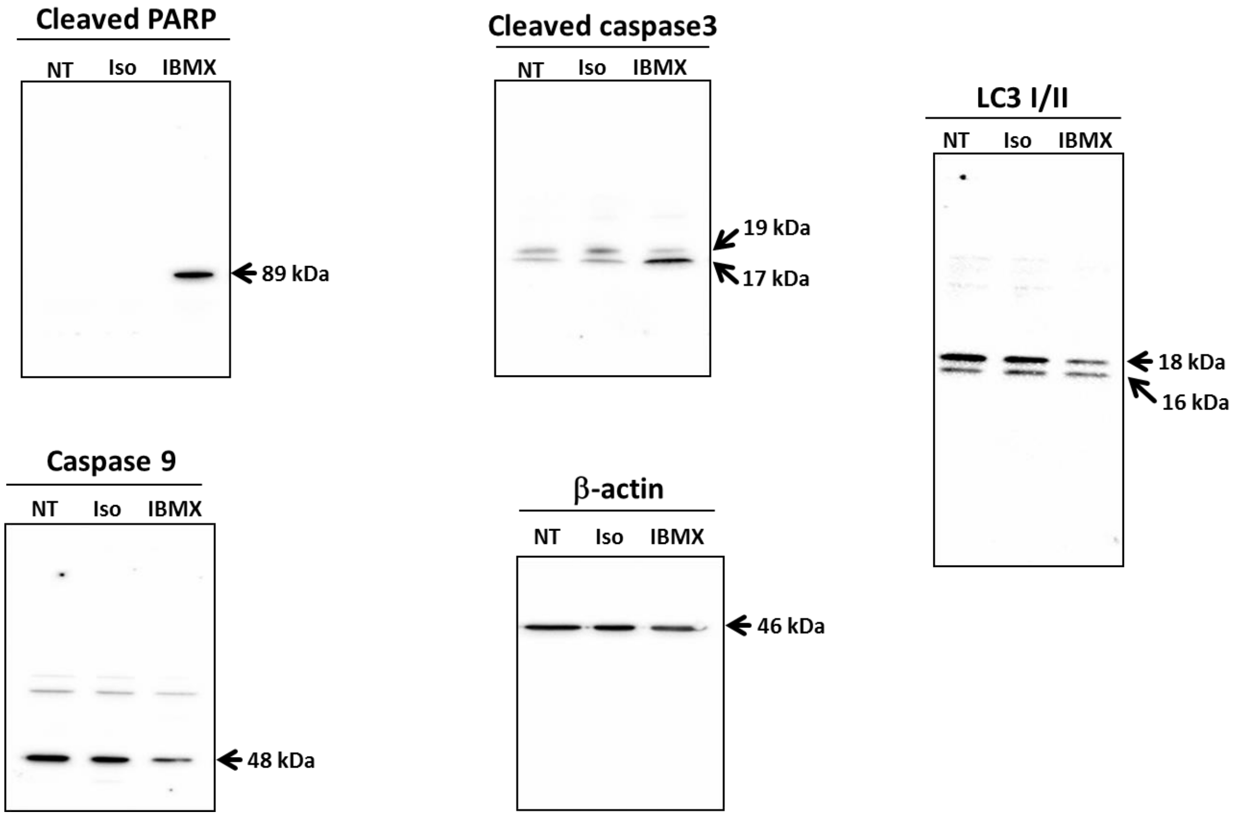

Supplementary Figure 5

Blots used to generate figure 4B

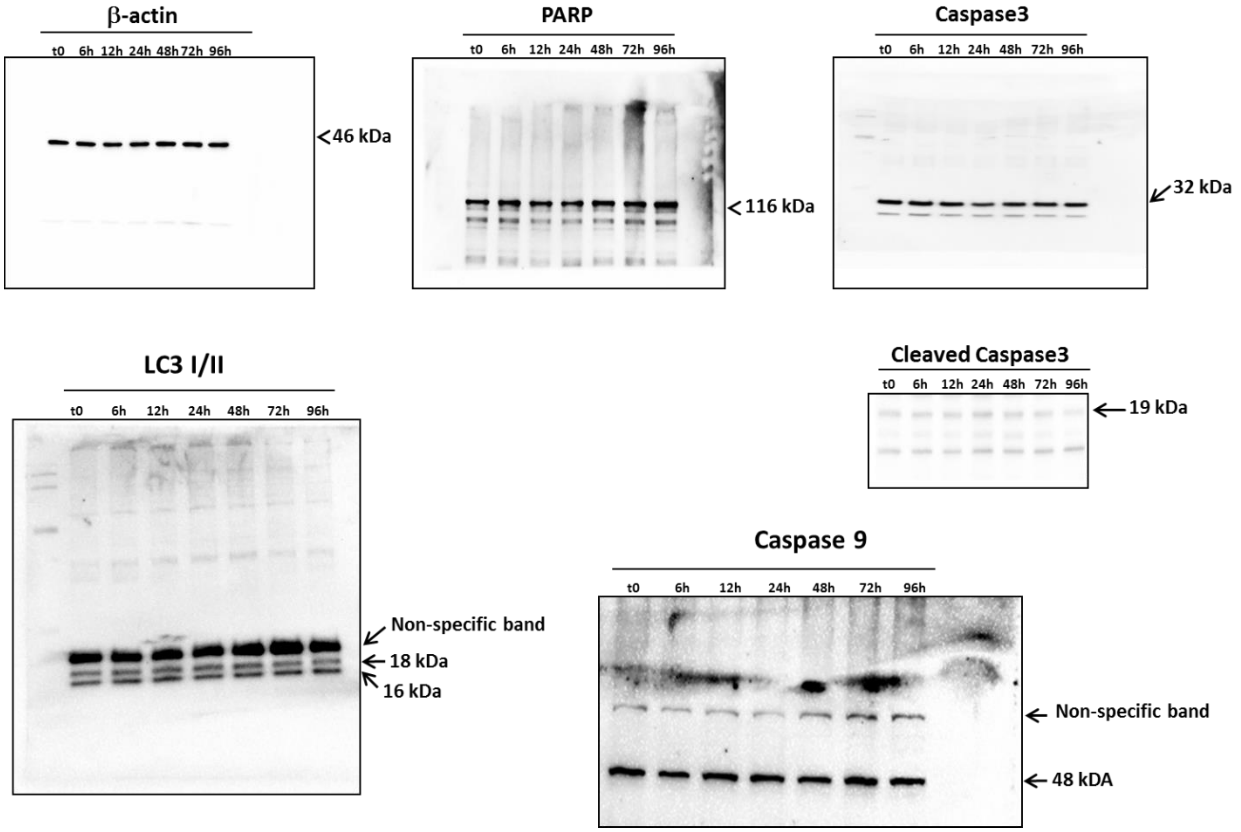

Supplementary Figure 6

Blots used to generate figure 4C

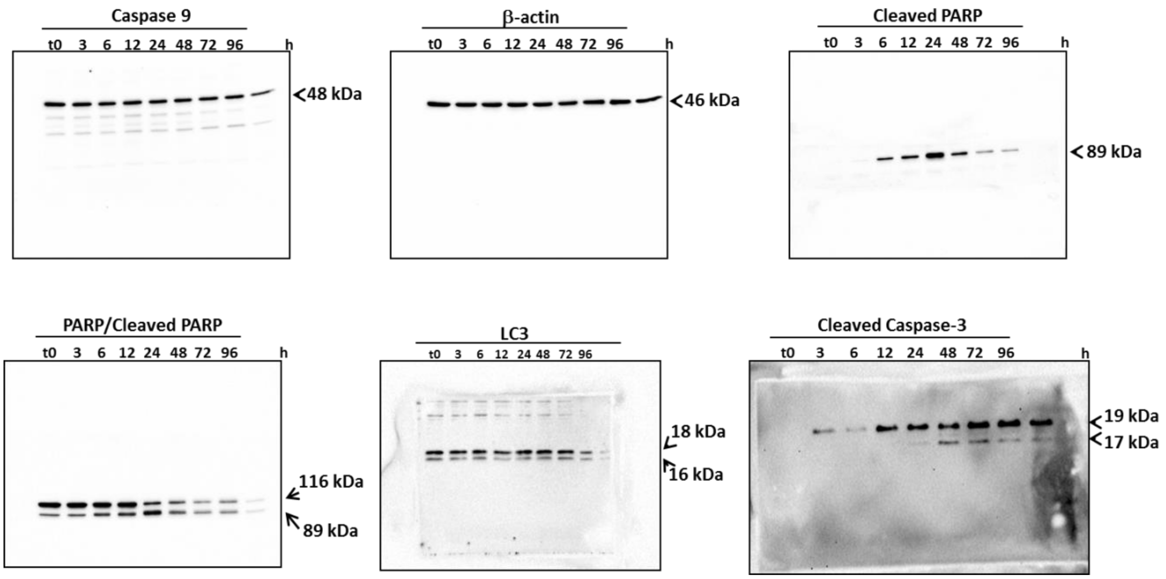

Supplementary Figure 7

Blots used to generate figure 5A and E

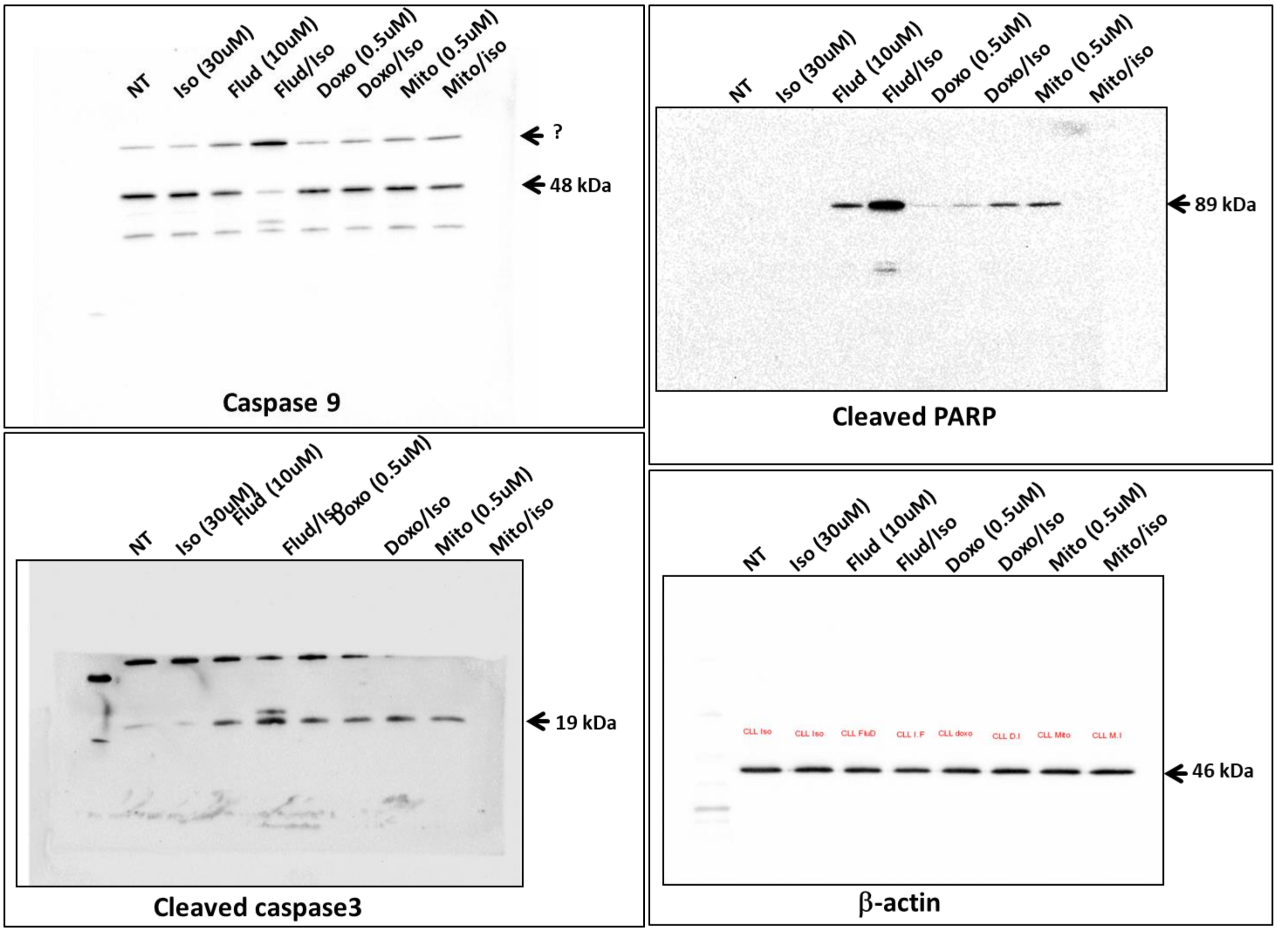

Supplementary Figure 8

Blots used to generate figure 6A

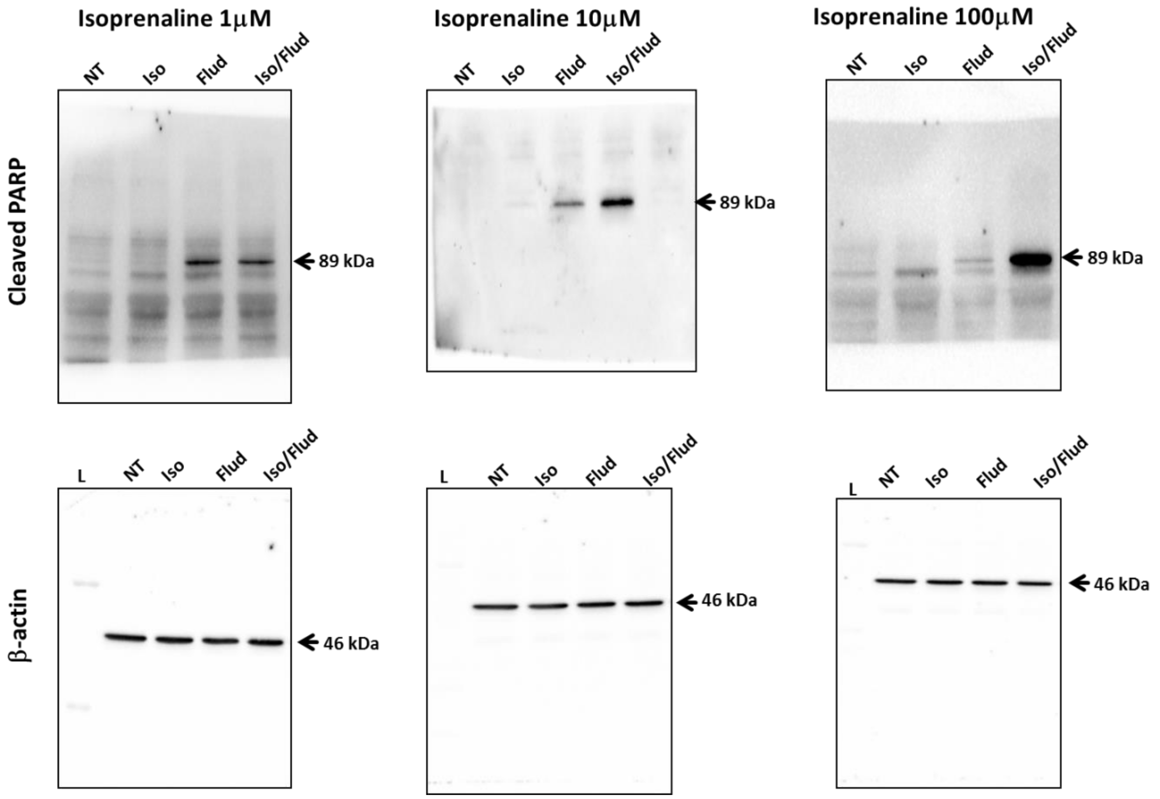

Supplementary Figure 9

Blots used to generate figure 7B

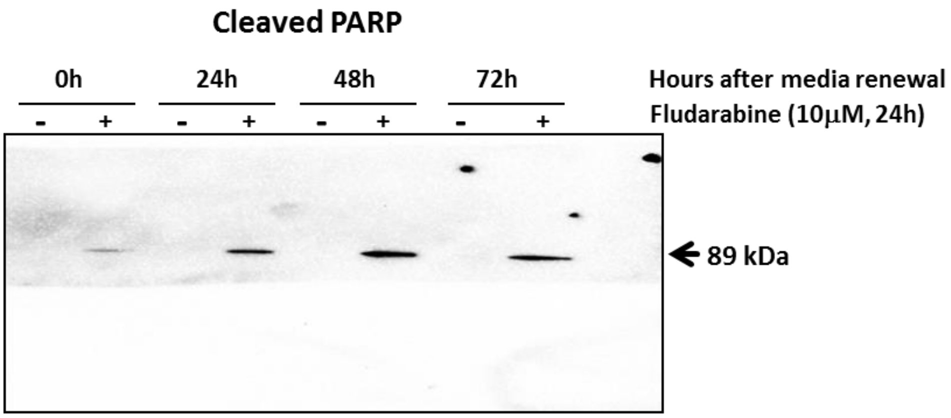

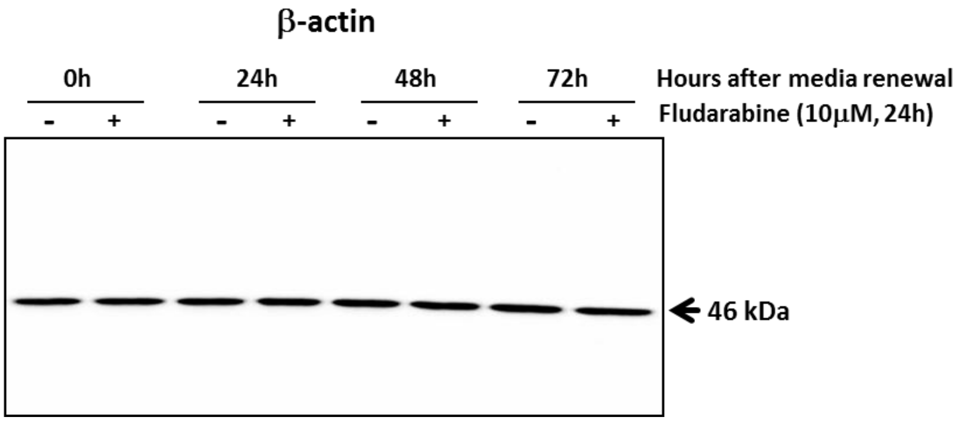

Supplementary Figure 10
